# Patterns of cognitive-emotional change after cognitive-behavioural treatment in emotional disorders: A 12-month longitudinal cluster analysis

**DOI:** 10.1371/journal.pone.0301746

**Published:** 2024-05-07

**Authors:** Sara Barrio-Martínez, Noelia Rodriguez-Perez, Amador Priede, Leonardo Adrián Medrano, Roger Muñoz-Navarro, Juan Antonio Moriana, María Carpallo-González, Maider Prieto-Vila, Paloma Ruiz-Rodríguez, Antonio Cano-Vindel, César González-Blanch

**Affiliations:** 1 Faculty of Psychology, Complutense University of Madrid, Madrid, Spain; 2 Valdecilla Biomedical Research Institute (IDIVAL), Santander, Spain; 3 Mental Health Centre, Hospital de Laredo, Laredo, Spain; 4 Pontificia Universidad Católica Madre y Maestra, Santiago de los Caballeros, Dominican Republic; 5 Department of Personality, Assessment and Psychological Treatments, Faculty of Psychology, University of Valencia, Valencia, Spain; 6 Department of Psychology, Universidad de Córdoba, Córdoba, Spain; 7 Maimónides Institute for Research in Biomedicine of Cordoba (IMIBIC), Córdoba, Spain; 8 Embarcaciones Primary Care Centre, Health Service of Madrid, Tres Cantos, Madrid, Spain; 9 Mental Health Centre, Marqués de Valdecilla University Hospital—IDIVAL, Santander, Spain; 10 Faculty of Health Sciences, Universidad Europea del Atlántico, Santander, Spain; Taipei Veterans General Hospital, TAIWAN

## Abstract

**Introduction:**

The aim of this study was to use cluster analysis based on the trajectory of five cognitive-emotional processes (worry, rumination, metacognition, cognitive reappraisal and expressive suppression) over time to explore differences in clinical and performance variables in primary care patients with emotional symptoms.

**Methods:**

We compared the effect of adding transdiagnostic cognitive-behavioural therapy (TD-CBT) to treatment as usual (TAU) according to cluster membership and sought to determine the variables that predicted cluster membership. 732 participants completed scales about cognitive-emotional processes, anxiety and depressive symptoms, functioning, and quality of life (QoL) at baseline, posttreatment, and at 12 months. Longitudinal cluster analysis and logistic regression analyses were carried out.

**Results:**

A two-cluster solution was chosen as the best fit, named as “less” or “more” improvement in cognitive-emotional processes. Individuals who achieved more improvement in cognitive-emotional processes showed lower emotional symptoms and better QoL and functioning at all three time points. TAU+TD-CBT, income level, QoL and anxiety symptoms were significant predictors of cluster membership.

**Conclusions:**

These results underscore the value of adding TD-CBT to reduce maladaptive cognitive-emotional regulation strategies. These findings highlight the importance of the processes of change in therapy and demonstrate the relevance of the patient’s cognitive-emotional profile in improving treatment outcomes.

## Introduction

Following the COVID-19 pandemic, mental health issues exponentially increased among the general population, reaching global prevalence rates of 26.9% for anxiety disorders and 28% for depression, respectively [1], becoming the two mental health conditions considered to be the largest contributors to global disability, with a major negative impact on quality of life (QoL) [2]. A wide variety of treatments have proven effective for emotional disorders, such as anxiety and depression, although the intervention with the most extensive empirical support for these disorders is cognitive-behavioural therapy (CBT) [3, 4]. Nevertheless, despite the high prevalence and economic costs of these disorders, and the use of evidence-based psychological treatments, most people with anxiety or depression disorders still do not respond adequately to treatment [5, 6]. The identification of processes underlying positive responses to therapy is necessary to develop most effective treatments and improve treatment outcomes [4]. This approach focuses on identifying mediators—variables that help to understand the underlying mechanism or process by which the independent variable affects the dependent variable—and moderators—variables that help to identify the individuals most likely to benefit from a given treatment—and on determining the mechanisms involved in the treatment response and in identifying which methods work better for different individuals.

In this regard, previous studies have demonstrated how individuals’ cognitive-emotional style can constitute a risk factor for developing these emotional disorders, exacerbating symptoms, and affecting daily functioning [7]. The cognitive-emotional processes that have received most attention to date are rumination, worry, metacognition, and emotion regulation [8–10]. In this sense, a previous study found that worry, rumination, negative metacognition and expressive suppression had a mediating role in the treatment of emotional disorders [11]. Moreover, it was observed that negative metacognition was a key mediator between TD-CBT and QoL [11]. A previous study established the moderating effect of cognitive reappraisal and expressive suppression on the effect of TD-CBT on treatment outcomes [12]. Specifically, individuals with higher levels of expressive suppression benefitted more from the addition of TD-CBT to treatment as usual (TAU) in terms of a greater reduction in anxiety and depressive symptoms versus TAU alone. We also showed that individuals with higher levels of cognitive reappraisal and expressive suppression at baseline obtained greater benefits in terms of QoL when psychological treatment was added to TAU.

In addition, several studies have demonstrated that processes such as rumination and worry are associated with poorer response to treatment, slower recovery, and a higher likelihood of relapse after treatment [13]. Kertz et al. (2015) described prototypical trajectories, suggesting that emotional symptoms were unlikely to improve without a decrease in processes such as rumination or worry [13]. Other authors have suggested that individuals with high levels of worry and rumination are less likely to benefit from CBT [14]. Nevertheless, these studies have important limitations, including limited evaluation time points (pre- and post-treatment) and a focus only on specific processes such as rumination or worry [13], thus leaving out other relevant cognitive-emotional processes and performance aspects (e.g., QoL or functioning). In addition, to our knowledge, no prior studies have evaluated the trajectory of certain cognitive-emotional processes together—rumination, worry, metacognition, expressive suppression and cognitive reappraisal—to establish differentiated cognitive-emotional profiles through longitudinal cluster analysis. Similarly, these processes have not been studied to identify risk factors and observe how the identified longitudinal clusters respond to different treatments. In this context, cluster analysis is key to understand the heterogeneity between different mental disorders in order to improve diagnostic criteria and our understanding about the processes of change associated with a better response to treatment [15].

In this context, we conducted a study to better understand the cognitive-emotional processes involved in emotional symptoms (mainly anxiety and depression). This study had three main aims. First, we aimed to identify clusters of individuals based on the trajectory of five cognitive-emotional processes (rumination, worry, metacognition, cognitive reappraisal and expressive suppression) over time to explore clinical and performance variables within a sample of primary care patients with emotional disorders. The second aim was to test the effect of the interaction between TD-CBT and cognitive-motional processes on clinical symptoms, QoL, and functioning. Finally, the third aim was to examine potential baseline predictors that determine membership in each cluster. Considering the close relationship between cognitive-emotional processes and symptoms of anxiety and depression, we hypothesized that participants belonging to the cluster that achieve more improvement in cognitive-emotional processes after treatment (i.e., lower levels of worry, rumination, negative metacognitive beliefs and expressive suppression, as well as higher levels of cognitive reappraisal at the different assessment points) would be associated with better clinical (anxiety and depressive symptoms) and performance levels (QoL and functioning) at all time points (pre-treatment, posttreatment, and after 12-months of follow-up). Additionally, we expected TD-CBT to be among the predictors of the cluster with a better cognitive-emotional response.

## Methods

### Participants

In the PsicAP study, 1061 individuals were randomly assigned to either the experimental arm (TAU+TD-CBT, N = 527) or the control arm (TAU alone, N = 534). This study focuses on 732 adult patients who had at least 2 assessment time points out of 3. Table 1 presents descriptive data for the sample, categorized by cluster membership based on the degree of improvement in cognitive-emotional processes.

**Table 1:** Baseline sociodemographic and clinical variables between cognitive-emotional clusters.

Variables	More improvement; n = 384^1^	Less improvement; n = 348^1^	95% CI Difference^2,3^	p-value^2^
Age at first visit	43.8 (11.2)	43.9 (11.8)	-1.8, 1.5	0.8
Gender, Male	78 (20%)	56 (16%)		0.14
Educational level, Basic studies	266 (69%)	259 (74%)		0.12
Intervention, Control	156 (41%)	205 (59%)		**<0.001**
Income level <24,000€	275 (72%)	294 (84%)		**<0.001**
Employment status, Not working	177 (46%)	169 (49%)		0.5
Marital status, Without a partner	123 (32%)	117 (34%)		0.6
PSWQ	27.3 (6.6)	33 (5.5)	-6.6, -4.8	**<0.001**
RRS	12.1 (3.2)	15 (3.2)	-3.4, -2.5	**<0.001**
MCQ	14.7 (3.8)	18.1 (3.4)	-4.0, -2.9	**<0.001**
ERQES	3.6 (1.4)	4.2 (1.5)	-0.8, -0.4	**<0.001**
ERQCR	4.3 (1.2)	4.1 (1.2)	-0.04, 0.3	0.12
WHOQOL	4.7 (1.4)	4.2 (1.3)	0.4, 0.7	**<0.001**
SDS	11.9 (7.3)	14.3 (7.7)	-3.5, -1.3	**<0.001**
GAD-7	11 (4.3)	13.8 (4.4)	-3.4, -2.1	**<0.001**
PHQ-9	12.4 (5)	15.1 (5.2)	-3.4, -1.9	**<0.001**

*Abbreviations =* ^1^ Mean (SD); n (%), ^2^ Welch Two Sample t-test, Pearson’s Chi-squared test, ^3^ *CI* = Confidence Interval. *More improvement =* More improvement in cognitive-emotional processes. *Less improvement =* Less improvement in cognitive-emotional processes. *PSWQ =* Penn State Worry Questionnaire. *RRS =* Ruminative Response Scale. *MCQ =* Metacognitive Questionnaire. *ERQES =* Expressive suppression strategy. *ERQCR =* Cognitive reappraisal strategy. *WHOQOL =* World Health Organization Quality of Life. *SDS =* Sheehan Disability Scale. *GAD-7 =* Generalized Anxiety Disorder-7. *PHQ-9 =* Patient Health Questionnaire-9. *NS =* Non-significant.

### Instruments

Ruminative thoughts were assessed using the Ruminative Response Scale (RRS) [16], a 5-item, self-report subscale corresponding to the "brooding" domain. Scores range from 5 to 20, with higher scores indicating a greater presence of rumination. Worry was assessed through the Penn State Worry Questionnaire- Abbreviated (PSWQ-A) [17], an 8-item, self-rated scale. Total scores range from 8 to 40, with higher scores indicating a greater presence of worry. Metacognition was measured with the Metacognitive Questionnaire-30 (MCQ-30) [18]. Only the 6-item corresponding to the "negative beliefs" domain were used. Scores range from 6 to 24, with higher scores indicating a greater presence of metacognitions. Emotion regulation strategies were determined according to the Emotion Regulation Questionnaire (ERQ) [19], a 10-item, self-report scale. Of these 10 items, six assess the cognitive reappraisal dimension (ERQCR) and the other four the expressive suppression dimension (ERQES). The ERQ scale ranges from 4–28 points for expressive suppression and from 6–42 points for cognitive reappraisal, with higher scores indicating a greater use of that emotion regulation strategy.

Anxiety symptoms were assessed using the GAD-7 [20], a 7-item, self-report scale in which total scores range from 0 to 21 (higher scores indicate a greater presence of anxiety symptoms). Depressive symptoms were assessed with the PHQ-9 [21], a 9-item, self-report scale with total scores ranging from 10 to 23 (higher scores indicate greater presence of depressive symptoms).

Quality of life (QoL) was measured through the World Health Organization Quality of Life scale (WHOQOL-BREF) [22], a 26-item, self-report scale. We converted each domain score into Z-score, and then summed and normalized these values to obtain a total score, which ranged from 0 to 10 (higher scores indicate greater perceived QoL). Functioning was assessed with the Sheehan Disability Scale (SDS) [23], a self-reported 5-item, 10-point Likert scale. The SDS assesses three main domains (work, family and social functioning) and two optional items (perceived stress and perceived social support). For purposes of the present study, we only considered the three main domains, which were summed to obtain a total score of global functional impairment (range: 0 to 30 points), with higher scores indicating worse individual functioning.

The following demographic variables were recorded: sex, age, marital status, educational level, employment status, and income level.

### Procedure

Data for this study were derived from the PsicAP study [24], a multicentre, two-arm, single-blind, randomized controlled trial (RCT), designed to evaluate the efficacy of adding TD-CBT to TAU versus TAU alone for treating emotional disorders in Spanish primary care centres [25]. Individuals with a diagnostic suspicion of an emotional disorder were invited by their general practitioner (GP) to participate in the study. Screening measures, including Generalized Anxiety Disorder-7 (GAD-7); the Patient Health Questionnaire-9 (PHQ-9); and the Patient Health Questionnaire-15 (PHQ-15) were used, and individuals exceeding cutoff points (≥ 10, ≥10, and ≥5, respectively) on at least one scale were included.

Participants who presented possible severe major depression (PHQ-9 score >24) and/or severe disability (Sheehan Disability Scale [SDS] score >25), underwent a semi-structured interview with a clinical psychologist to rule out the presence of any severe mental disorder or recent suicidal behaviour. Other exclusion criteria included intellectual disability, insufficient Spanish language skills, and concurrent psychological therapy. All individuals who did not meet the inclusion criteria were referred to their GP for alternative treatment.

All participants were randomized in a 1:1 ratio to either the experimental arm (TAU+TD-CBT) or control group (TAU alone). The experimental group received seven, 90-minute sessions of TD-CBT in groups of 8–10 participants over a 3–4-month period. The sessions were provided by clinical psychologist and involved behavioural techniques, relaxation, cognitive restructuring, and relapse prevention. TAU involved regular consultations with the treating GP, who assessed the patient’s physical and psychological symptoms. In general, the treatment involved the prescription of psychopharmacological medications (anxiolytics, antidepressants or hypnotics) in accordance with the GP criteria, and/or informal counselling/support provided during brief consultations lasting approximately 10 minutes [25, 26].

The authors assert that all procedures contributing to this work comply with the ethical standards of the relevant national and institutional committees on human experimentation and with the Helsinki Declaration of 1975, as revised in 2008. The present study constitutes a secondary analysis based on a previous registered clinical trial (ISRCTN58437086) and was approved by the Clinical Research Ethics Committee of Primary Care of the Valencian Region in 2013 (code: 2013-001955-11). Written informed consent was obtained from all patients, and those who agreed to participate were provided with an information sheet containing all the details of the study.

### Data analysis

K means clustering of longitudinal data analysis was performed with the kml3d package for R. This method allows to explore the joint temporal evolution of five cognitive-emotional processes (PSWQ, RRS, MCQ, ERQES, ERQCR) across 3 time points to identify homogeneous clusters of cognitive-emotional outcome trajectories. Patients with at least 2 assessment time points were retained in the analysis (N = 732), with missing values were imputed by copyMean method. The multidimensional Euclidean metrics was used to calculate interindividual distances needed to build the clusters. The number of optimal clusters was determined based on Calinski-Harabatz [27] and Davies Bouldin [28] indices, selecting the clustering solution that minimized the proximity indicator (average within-cluster distance/between-cluster distance). G*Power 3.1 software [29] was used to perform the power analysis, which showed that, with our sample size (n = 732), we had sufficient power to detect large effect sizes with our analyses (i.e., a statistical power of 0.999 (1-β = 0.999), an effect size of 0.8, and a significance level of 0.05).

Clinical and performance differences by cluster were evaluated using Chi-square and Student’s t test. Effect sizes for these analyses were calculated using Cohen’s d. Logistic regression analysis was also performed using the lme4 package, which is a widely use tool in R for fitting linear mixed effects models. It provides a flexible framework for analysing complex hierarchical and longitudinal data structures. This analysis aimed to identify the baseline variables that predicted the trajectories of the five cognitive-emotional processes. We included as predictors the baseline variables in which significant differences were obtained between the two clusters (i.e., experimental treatment [TAU+TD-CBT], income level, QoL, functioning and anxiety and depressive symptoms). We calculated the odds ratios from the model estimate coefficients along with their 95% confidence intervals. All the assumptions of the model were fulfilled. All statistical analyses used a two-tailed α level of 0.05. All tests were carried out in R software (script available upon request).

## Results

No significant differences were observed between dropouts (n = 328, 30.9%) and individuals in the analysis (n = 732, 69.1%) in any of the variables analysed (p> 0.05).

### Longitudinal trajectory of cognitive-emotional processes

#### Description of the clusters

The two-cluster solution was chosen as the best fit based on the cluster analysis performed (S1 Fig). The sample was divided into two groups according to the profile found (i.e., cognitive-emotional outcome trajectories): more improvement in cognitive-emotional processes (n = 384); and less improvement in cognitive-emotional processes (n = 348) (Tables 1 and 2). The participants with a more improvement in cognitive-emotional processes showed lower levels of worry, rumination, metacognition and expressive suppression than those who showed less improvement in cognitive-emotional processes at the different assessment points. However, significant between-group differences in cognitive reappraisal were observed only at the posttreatment evaluation and thereafter.

**Table 2:** Posttreatment and 12-month follow-up clinical and performance variables differences between cognitive-emotional clusters.

Posttreatment					12-month follow-up			
Variables	More improvement; n = 384^1^	Less improvement; n = 348^1^	95% CI Difference^2,3^	p-value^2^	More improvement; n = 384^1^	Less improvement; n = 348^1^	95% CI Difference^2,3^	p-value^2^
WHOQOL	5.14 (1.75)	3.64 (1.72)	1.2, 1.8	**<0.001**	7.36 (1.04)	6.25 (1.04)	0.89, 1.3	**<0.001**
SDS	7.22 (6.99)	12.71 (8.07)	-6.7, -4.3	**<0.001**	5.87 (7.07)	12.92 (8.57)	-8.7, -5.5	**<0.001**
GAD-7	5.45 (3.94)	11.21 (5.13)	-6.5, -5.0	**<0.001**	4.14 (3.70)	10.90 (5.59)	-7.7, -5.8	**<0.001**
PHQ-9	6.35 (4.62)	12.70 (6.42)	-7.2, -5.5	**<0.001**	5.48 (4.66)	11.81 (6.63)	-7.5, -5.2	**<0.001**

*Abbreviations =* ^1^ Mean (SD); n (%), ^2^ Welch Two Sample t-test, Pearson’s Chi-squared test, ^3^ *CI* = Confidence Interval. *More improvement =* More improvement in cognitive-emotional processes. *Less improvement =* Less improvement in cognitive-emotional processes. *WHOQOL =* World Health Organization Quality of Life. *SDS =* Sheehan Disability Scale. *GAD-7 =* Generalized Anxiety Disorder-7. *PHQ-9 =* Patient Health Questionnaire-9. *NS =* Non-significant.

Individuals with more improvement in cognitive-emotional processes from baseline to 12 months follow-up showed large effect sizes in worry, rumination and metacognition (d = 1.00, d = 0.98, and d = 1.00 respectively). Expressive suppression and cognitive reappraisal strategies showed small to medium effect sizes (d = 0.41 and d = -0.33, respectively). Conversely, individuals with less improvement in cognitive-emotional processes exhibited small effect sizes for worry, rumination, metacognition, expressive suppression, and cognitive reappraisal (d = 0.26, d = 0.30, d = 0.27, d = -0.01, and d = -0.01, respectively).

### Sociodemographic, clinical and performance differences between clusters

A higher percentage of individuals who achieved more improvement in cognitive-emotional processes had a high income level and were more likely to belong to the experimental group at baseline (see Table 1). About clinical and performance variables, the trajectories were maintained at all three time points. Individuals with more improvement in cognitive-emotional processes had lower emotional symptoms and better QoL and functioning at baseline, posttreatment and the 12-month follow-up compared to participants with less improvement in cognitive-emotional processes (Tables 1 and 2), even though both groups improved in terms of clinical symptoms and performance.

### Effect of TD-CBT on cluster membership

With regards to the effect of the experimental group on treatment outcomes, among individuals with less improvement in cognitive-emotional processes at the posttreatment assessment, participants who received TAU+TD-CBT obtained better results in metacognition, cognitive reappraisal, functioning, and emotional symptoms than controls (TAU alone). However, these differences were maintained at the 12-month follow-up assessment only for metacognition and functioning. The experimental treatment also influenced treatment outcomes in individuals with more improvement in cognitive-emotional processes. At the posttreatment evaluation, the experimental group obtained greater improvement in numerous variables—worry, cognitive reappraisal, QoL, functioning, and anxiety and depressive symptoms—compared to the control group. These differences were maintained at the 12-month assessment (Table 3).

**Table 3:** Differences according to the type of intervention administered in cognitive, emotional, clinical and performance variables between individuals with more improvement in cognitive-emotional processes and less improvement in cognitive-emotional processes.

Less improvement		Posttreatment			12-month follow-up			
	TAU (N = 205)^1^	TAU+TD-CBT (N = 143)^1^	95%CI Difference^2,3^	p-value^2^	TAU (N = 205)^1^	TAU+TD-CBT (N = 143)^1^	95%CI Difference^2,3^	p-value^2^
PSWQ	32.6 (5.3)	31.5 (5.4)	-0.22, 2.3	0.1	31.9 (6)	31 (5.2)	-0.7, 2.7	0.3
RRS	14.9 (3.4)	14.7 (3.6)	-0.71, 1.0	0.8	14.4 (3.6)	13.5 (3.4)	-0.2, 2.0	0.091
MCQ	18.2 (3.5)	17.2 (3.7)	0.21, 1.9	**0.015**	17.8 (4.2)	16.4 (3.9)	0.1, 2.6	**0.030**
ERQES	4.2 (1.6)	4.1 (1.4)	-0.2, 0.5	0.5	4.3 (1.5)	4.1 (1.5)	-0.2, 0.7	0.3
ERQCR	4 (1.4)	4.5 (1.3)	-0.8, -0.2	**0.003**	4.1 (1.2)	4.2 (1.2)	-0.4, 0.4	0.9
WHOQOL	3.5 (1.8)	3.9 (1.6)	-0.8, 0.02	0.065	6.2 (1.0)	6.3 (1.1)	-0.4, 0.2	0.6
SDS	13.7 (8.2)	11.1 (7.6)	0.8, 4.5	**0.005**	14.2 (8.2)	11.4 (8.8)	0.2, 5.4	**0.036**
GAD-7	12.8 (4.9)	8.7 (4.6)	3, 5.2	**<0.001**	11.6 (5.6)	10 (5.5)	-0.1, 3.3	0.057
PHQ-9	14.3 (6.5)	10.2 (5.4)	2.7, 5.5	**<0.001**	12.5 (6.7)	10.9 (6.5)	-0.4, 3.6	0.11
More improvement	Posttreatment				12-month follow-up			
	TAU (N = 156)^1^	TAU+TD-CBT (N = 228)^1^	95%CI Difference^2,3^	p-value^2^	TAU (N = 156)^1^	TAU+TD-CBT (N = 228)^1^	95%CI Difference^2,3^	p-value^2^
PSWQ	23.6 (6.2)	21.2 (60.1)	1.0, 3.7	**<0.001**	21.9 (6.6)	20 (6.4)	0.1, 3.7	**0.039**
RRS	10.1 (2.8)	9.9 (2.9)	-0.4, 0.8	0.6	9.4 (2.7)	9 (2.9)	-0.3, 1.2	0.2
MCQ	11.9 (3.3)	11.4 (3.2)	-0.2, 1.2	0.2	11.4 (3.8)	10.7 (3.5)	-0.2, 1.8	0.13
ERQES	3.1 (1.3)	3.2 (1.3)	-0.4, 0.2	0.6	3 (1.3)	3. (1.3)	-0.3, 0.4	0.9
ERQCR	4.4 (1.3)	4.9 (1.1)	-0.8, -0.3	**<0.001**	4.3 (1.4)	5 (1.2)	-1.0, 0.3	**<0.001**
WHOQOL	4.7 (1.7)	5.5 (1.7)	-1.1, -0.4	**<0.001**	7.1 (1)	7.6 (1)	-0.8,-0.2	**<0.001**
SDS	8.1 (7.1)	6.6 (6.9)	0.01, 3.1	**0.048**	7.9 (8.1)	4.6 (6)	1.3, 5.3	**0.002**
GAD-7	6.9 (4.4)	4.5 (3.3)	1.6, 3.3	**<0.001**	5.4 (4)	3.4 (3.3)	1.0, 3.0	**<0.001**
PHQ-9	7.9 (4.9)	5.3 (4.2)	1.6, 3.6	**<0.001**	6.6 (4.6)	4.8 (4.6)	0.6, 3.1	**0.004**

*Abbreviations =* ^1^ Mean (SD); n (%), ^2^ Welch Two Sample t-test, Pearson’s Chi-squared test, Fisher’s exact test, ^3^*CI* = Confidence Interval. *More improvement =* More improvement in cognitive-emotional processes. *Less improvement =* Less improvement in cognitive-emotional processes. *PSWQ =* Penn State Worry Questionnaire. *RRS =* Ruminative Response Scale. *MCQ =* Metacognitive Questionnaire. *ERQES =* Expressive suppression strategy. *ERQCR =* Cognitive reappraisal strategy. *WHOQOL =* World Health Organization Quality of Life. *SDS =* Sheehan Disability Scale. *GAD-7 =* Generalized Anxiety Disorder-7. *PHQ-9 =* Patient Health Questionnaire-9. *TAU* = Treatment as usual. *TD-CBT =* transdiagnostic cognitive-behavioural therapy. *NS =* Non-significant.

### Predictors of cluster membership

TAU+TD-CBT, higher income level, greater QoL, and fewer anxiety symptoms at baseline were significant predictors of membership in the more improvement cluster (Table 4). Although there were significant differences between the two clusters in functioning and depressive symptoms (Table 1) in the bivariate analyses, these variables did not predict membership in the clusters once the effects of the other clinical, sociodemographic and performance variables were considered simultaneously in the logistic regression.

**Table 4:** Logistic regression examining cluster membership potential predictors.

Variables	OR (95% CI)	p-value
TAU+ TD-CBT	1.6 (1.4–1.9)	**<0.001**
Income level	1.4 (1.1–1.6)	**<0.001**
WHOQOL	1.2 (1–1.5)	**<0.05**
SDS	0.9 (0.7–1)	0.2
GAD-7	0.6 (0.5–0.7)	**<0.001**
PHQ-9	0.9 (0.7–1.1)	0.1

*Abbreviations*: *OR =* Odds ratio.*CI* = Confidence Interval. *TAU+TD-CBT =* Transdiagnostic cognitive-behavioural therapy plus treatment as usual. *WHOQOL =* World Health Organization Quality of Life. *SDS =* Sheehan Disability Scale. *GAD-7 =* Generalized Anxiety Disorder-7. *PHQ-9 =* Patient Health Questionnaire-9. *NS =* Non-significant.

## Discussion

In the present study, we compared the longitudinal course of five cognitive-emotional processes—rumination, worry, metacognition, expressive suppression and cognitive reappraisal—in individuals with depressive and anxiety symptoms. We obtained two clearly differentiated clusters based on the cognitive-emotional trajectories, which we categorized as either a more or less improvement in cognitive-emotional processes. We have also observed that TAU+TD-CBT was a potential predictor that determine membership in the “more improvement” cluster.

At baseline, posttreatment and 12-month follow-up assessments, individuals who demonstrated more improvement in cognitive-emotional processes were characterized by lower levels of worry, rumination, metacognition, and expressive suppression. Moreover, the effect sizes for worry, rumination and metacognition were all large (d>.98), with small to medium effect sizes (d>0.33) in expressive suppression and cognitive reappraisal strategies between baseline and the 12-month follow-up. However, significant between-group differences in cognitive reappraisal were only observed in the posttreatment period, with an increased use of this strategy only in the more improvement cluster. This between-group difference was maintained at the 12-month follow-up. Changes in this emotion regulation strategy after treatment may be due to the fact that a higher percentage of people in this cluster received TD-CBT, where they worked on different aspects such as cognitive restructuring, relaxation techniques or psychoeducation [30, 31], on which TD-CBT has a direct effect [11].

Although individuals in the less improvement cluster also showed longitudinal improvements in cognitive-emotional processes, the effect sizes were significantly smaller (d<0.30). It seems likely that CBT played a role in improving these cognitive-emotional processes, albeit to a lesser extent than in the patients in the more improvement cluster, in which a higher proportion of individuals had received TAU+TD-CBT.

In terms of the sociodemographic differences between the two clusters, individuals in the more improvement cluster had higher income levels at baseline. This finding is consistent with other studies, which have already established that individuals with lower incomes have more worry, rumination [32] and more deficits in emotion regulation [33], which a negative repercussion on well-being and mental health.

As expected, all the participants who received TAU+TD-CBT had better treatment outcomes (clinical symptoms and performance) than those who only received TAU alone. This finding is congruent with previous studies that have shown that CBT improves treatment outcomes in the short, medium and long term [24, 31]. In fact, CBT is the most empirically validated intervention for emotional disorders [3, 4], significantly improving QoL [34] and functioning [24].

Individuals who had more improvement in cognitive-emotional processes showed fewer emotional symptoms and higher levels of QoL and functioning at the baseline, posttreatment and 12-month follow-up assessments. At the posttreatment evaluation, individuals who received TAU+TD-CBT and had a more improvement in cognitive-emotional processes had better results than those who received TAU alone in terms of worry, cognitive reappraisal, QoL, functioning, and anxiety and depressive symptoms. Moreover, these differences were maintained at the 12-month follow-up. The notable positive response to psychological treatment in participants in this cluster shows that not only do cognitive-emotional processes act as predictors of response [13, 35–38], but also that individuals with a more adaptative cognitive-emotional profile who received CBT would be more likely to have a better understanding, thus increasing responsiveness to treatment [39].

Surprisingly, rumination did not differ for either cluster, based on the type of intervention offered. This suggests that TD-CBT might not have directly targeted rumination or that additional factors may have influenced its effectiveness in reducing negative thought patterns. In the same way, unexpectedly, we also observed that negative metacognitive beliefs improved in the less improvement cluster but not in the more improvement cluster. This outcome is likely attributable to the fact that individuals belonging to the more improvement cluster may already possess more adaptive cognitive strategies, which could result in a diminished impact of the experimental treatment on these beliefs. Consequently, this could explain the absence of significant differences by adding TD-CBT to TAU, compared to TAU alone in the more improvement cluster.

We observed that anxiety symptoms, lower income levels and worse perceived QoL acted as risk factors for worse cognitive-emotional progression over time and thus, a greater probability of a decreased response to treatment. Therefore, these factors should be considered when administering psychological treatment to patients, since they play a key role in achieving optimal results and a more durable recovery. Psychological treatment was also a predictor of belonging to the cluster with more improvement in cognitive-emotional processes. Previous studies have demonstrated the efficacy of CBT in improving clinical symptoms [3, 24]; however, previous literature only analyse the evolution of some of these cognitive-emotional processes separately [40]. Knowing the different trajectories that follow all these cognitive-emotional processes after TAU+TD-CBT depending on the cluster membership is a great advance, since one of the aims of CBT is to improve cognitive-emotional processes.

Our study has several limitations. Firstly, the examination of cognitive-emotional trajectories was limited to the short term (5–16 months, from baseline to the 12-month follow-up), and the analysis was confined to three specific time points, rendering long-term changes uncertain. Second, this patient sample consisted of individuals with mild to moderate emotional disorders symptoms, which means we must be cautious before generalizing the results. A third limitation is the use of self-reported scales, even though all of these scales are well-validated measures in primary care settings [21, 41, 42]. Finally, the high attrition rate in this study could have influenced the results obtained. However, no significant differences were found when comparing individuals who completed at least two of three assessments versus those who did not. Moreover, similar high attrition rates have been described in other RCTs carried out in primary care settings [43].

In conclusion, we identified two distinct trajectories based on the patients’ cognitive-emotional profile. Individuals who experienced more improvement in cognitive-emotional processes had better outcomes after treatment, both in the immediate posttreatment assessment and after 12-months of follow-up. These findings underscore the value of adding TD-CBT to TAU for the treatment of primary care patients. These results highlight that this psychological intervention not only enhances and sustains the positive treatment effects, but also augments the likelihood of greater improvements in cognitive-emotional processes, even in cases where individuals experience reduced benefits due to high initial levels of these processes. In situations where the benefit margin is limited, it may be advisable within clinical practice to consider a more tailored treatment approach, focusing specifically on the most maladaptive cognitive-emotional processes, as opposed to a more generalized group therapy approach. Moreover, these findings highlight the value of processes of change in therapy thus providing more evidence to support the value of personalized treatments.

## Supporting information

S1 Fig Plots of Calinski-Harabatz and Davies Bouldin indexes for 2–6 clusters on cognitive variables (PSWQ, RRS, MCQ, ERQES and ERQCR). (DOCX)
