# Supplementary material for: Patterns of cognitive-emotional change after cognitive-behavioural treatment in emotional disorders: A 12-month longitudinal cluster analysis
Source: PLoS One. 2024 May 7;19(5):e0301746. doi: 10.1371/journal.pone.0301746 (PMC11075866; doi:10.1371/journal.pone.0301746)
Supplement: S1 Fig — (DOCX) [file pone.0301746.s001.docx]

**S1 Fig.** Plots of Calinski-Harabatz and Davies Bouldin indexes for 2-6 clusters on cognitive variables (PSWQ, RRS, MCQ, ERQES and ERQCR).


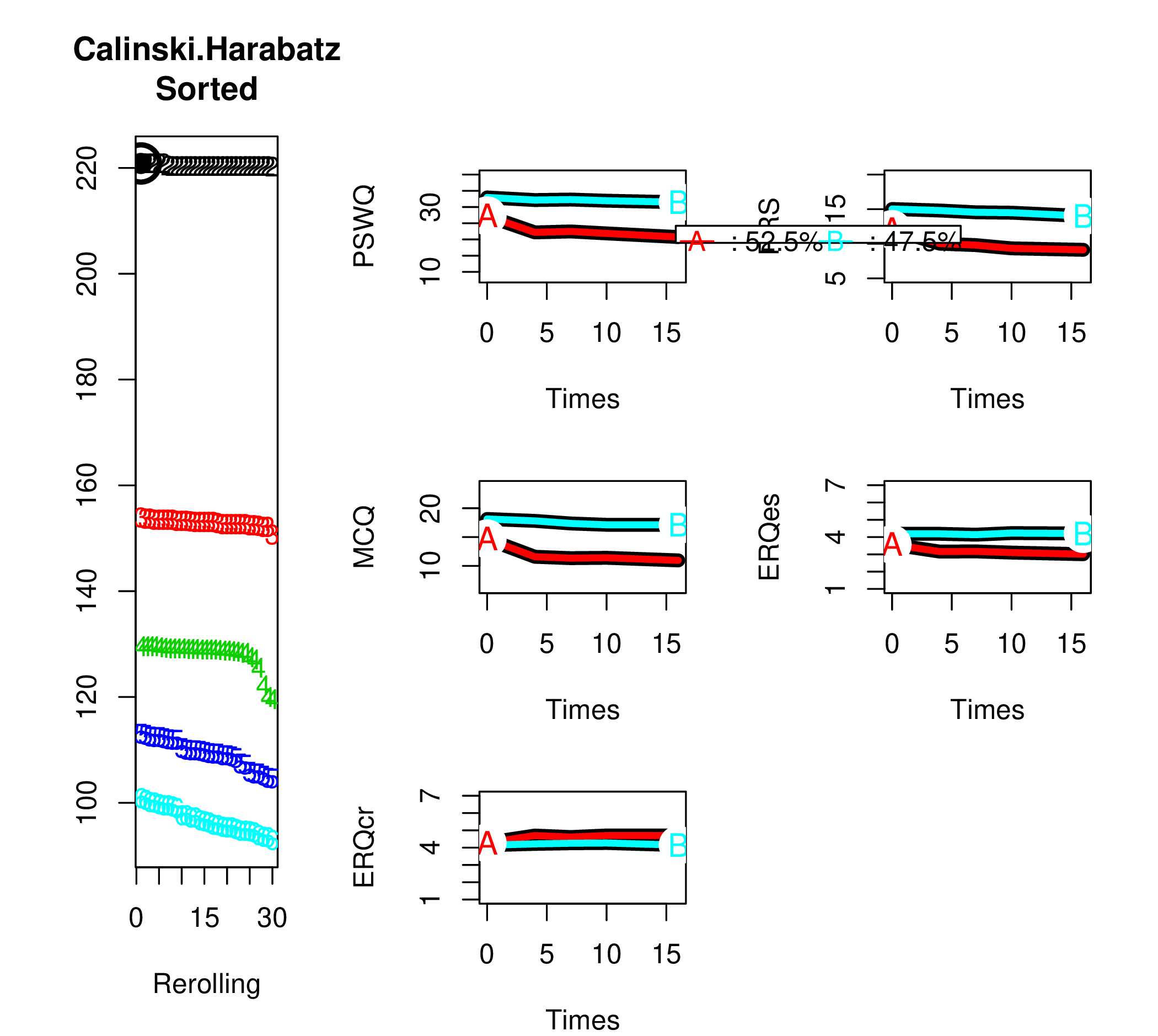


**
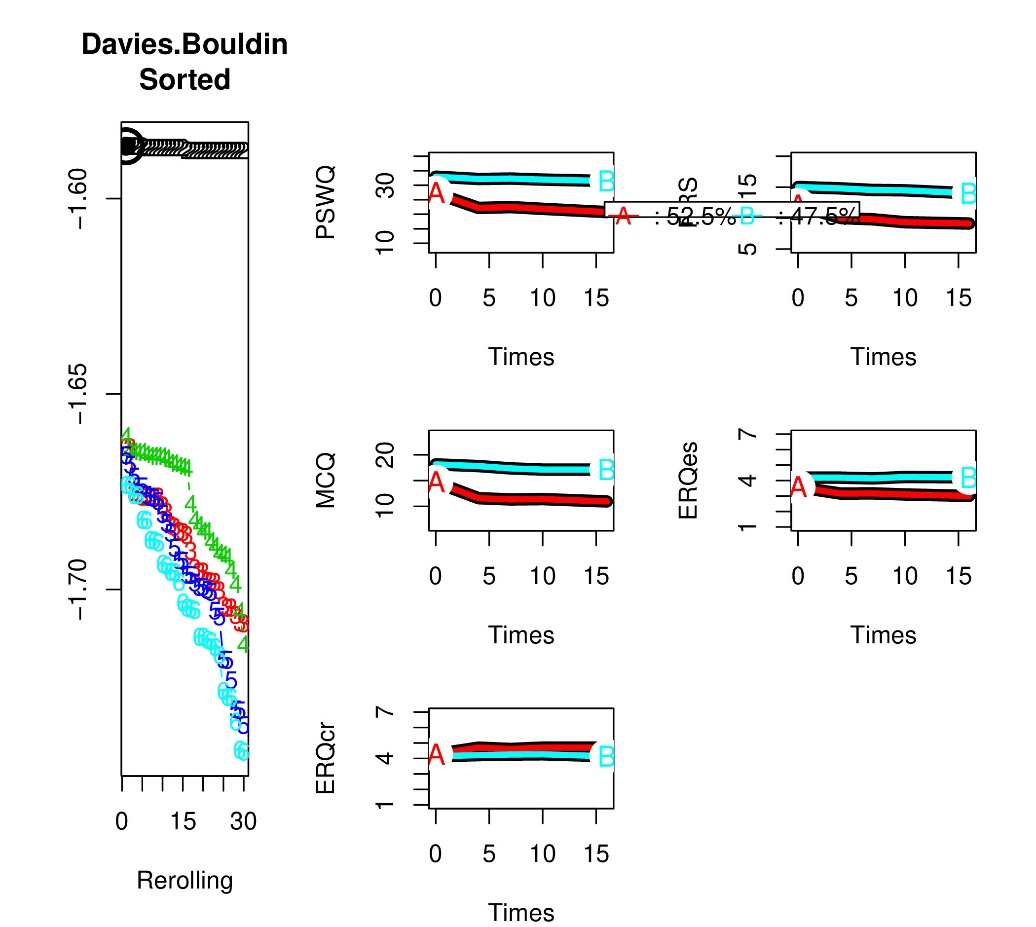
**
